# Jellyfish stings‐induced cardiac failure was ameliorated through AAG‐mediated glycogen‐driven ATP production

**DOI:** 10.1002/EXP.20230089

**Published:** 2024-02-20

**Authors:** Zhen Qin, Jinhong Chen, Fang Liu, Bingbing Li, Chenchen Zhang, Xiuxiu Wang, Lin Liu, Mingke Wang, Tingfang Wang, Su Wang, Feifei Yu, Shifeng Wang, Jishun Yang

**Affiliations:** ^1^ PLA Naval Medical Center Naval Medical University (Second Military Medical University) Shanghai China; ^2^ School of Health Sciences and Engineering University of Shanghai for Science and Technology Shanghai China; ^3^ School of Medicine Shanghai University Shanghai China; ^4^ Center for Translational Neuromedicine University of Rochester Medical Center Rochester New York USA

**Keywords:** cardiac function, glycolytic/mitochondrial metabolic switches, jellyfish stings, therapeutic target

## Abstract

Jellyfish stings have become a common injury among fishermen and divers. Severe jellyfish stings could worsen cardiac function and even cause cardiac complications, ultimately leading to cardiac failure (CF). Currently, there are no effective drugs available. Single cell sequencing revealed alpha‐1 acid glycoprotein (AAG), an energy regulatory protein targeting to glycogen, was highly expressed in jellyfish stings‐induced CF patients. However, the mechanism remains elusive. It is postulated that AAG could increase glycogen metabolism, protecting against jellyfish stings‐induced CF. AAG deletion exacerbated CF, while exogenous and endogenous AAG ameliorated CF. AAG also rescued the decline triggered by the AAG knockout (KO). Intriguingly, AAG improved cardiac function and metabolic adaptation by glycogen‐driven ATP production, shifting mitochondrial/glycolytic ATP production towards glycolysis. Sorted by single‐cell RNA sequencing and spatial transcription technology, CC‐chemokine receptor 5 (CCR5) and Peroxisome proliferator‐activated receptor‐gamma coactivator‐1alpha (PGC‐1α) were differentially expressed. Mechanistically, CCR5 inhibitor MVC abolished AAG's protective effect and PGC‐1α overexpression. Collectively, jellyfish stings‐induced CF was ameliorated through AAG‐mediated glycogen‐driven ATP production, promoting glycolytic/mitochondrial metabolic switches to rely energetically primarily on glycolysis, which might serve as a therapeutic target of CF.

## Graphical abstract

Single cell sequencing revealed AAG, an energy regulatory protein targeting to glycogen, was highly expressed in jellyfish stings‐induced CF patients. We postulated AAG could increase glycogen metabolism, protecting against jellyfish stings‐induced CF. Intriguingly, AAG improved cardiac function and metabolic adaptation by glycogen‐driven ATP production, shifting mitochondrial / glycolytic ATP production towards glycolysis. Sorted by single‐cell RNA sequencing and spatial transcription technology, CCR5 and PGC‐1α were differentially expressed. Collectively, jellyfish stings‐induced CF was ameliorated through AAG‐mediated glycogen‐driven ATP production, promoting glycolytic / mitochondrial metabolic switches to rely energetically primarily on glycolysis, which might serve as a therapeutic target of CF.

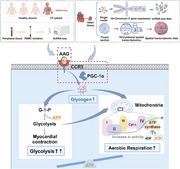

## 1 INTRODUCTION

Jellyfish stings are the commonest marine animal injuries worldwide, with approximately 150 million envenomation cases annually. Human beings often become victims accidentally while swimming, diving or fishing. Contact with the jellyfish tentacles triggers the explosive discharge of stinging capsules that inject extremely potent and rapidly acting venom into the victim or prey. While localized symptoms such as skin allergies and itching are well‐documented, severe systemic cases, including life‐threatening cardiac complications, have also been reported.^[^ ^1^ ^]^ Notably, cardiovascular damage is the main cause of serious consequences, even death, induced by jellyfish stings,^[^ ^1^, ^2^ ^]^ in which acute total cardiac failure (CF) represents a potentially life‐threatening occurrence and is the basic physiopathologic change.^[^ ^3^ ^]^ However, current treatment options are limited and often focus on symptomatic relief rather than targeting the underlying molecular mechanisms.

Jellyfish venoms are bioactive protein mixtures that could disrupt cellular processes and energy metabolism, leading to cardiac dysfunction. Intriguingly, exposure to jellyfish venoms can trigger a cascade of events leading to cardiac dysfunction, including impaired contraction and energy deprivation.^[^ ^4^, ^5^ ^]^ Cardiac metabolism and energy supply disorders contribute to the progression of CF.^[^ ^6^ ^]^ As a metabolically biological pump, the heart is an organ with highly demanding energy and consumes nearly 10% of the whole‐body's fuel, over seven times its weight in ATP daily.^[^ ^7^, ^8^ ^]^ Although ATP production depends on oxidative phosphorylation (OXPHOS) by the electron transport chain (ETC) in mitochondria,^[^ ^9^, ^10^ ^]^ cardiomyocytes are uniquely flexible in utilizing metabolic substrates to maintain ATP production and meet energy needs. Therefore, a promising strategy for metabolic intervention in CF is to modulate substrate utilization and energy supply.^[^ ^11^ ^]^

One important aspect of cellular energy metabolism is the regulation of glycogen, a major endogenous form of energy storage and utilization, including in the heart. Glycogen serves as a readily available source of glucose, enabling ATP production through glycolysis during increased energy demands. Disruption of glycogen metabolism could compromise energy production and impair cardiac function.^[^ ^12^, ^13^, ^14^, ^15^ ^]^ Specifically, glycogen is an important energy substrate for the aerobic heart when the workload is acutely increased,^[^ ^16^ ^]^ and the main energy source for the heart during hypoxia.^[^ ^17^ ^]^ A total of 60–70% of the glucose taken up by the heart was incorporated into glycogen.^[^ ^14^, ^15^ ^]^ Recent data suggest that glycogen‐derived glucose is preferentially oxidized compared with exogenous glucose, while improper glycogen processing is a key feature of cardiac metabolic stress.^[^ ^15^, ^18^, ^19^ ^]^ Moreover, glycogen synthase (GS) 1 deletion reduced cardiac glycogen storage and impaired cardiac function.^[^ ^20^ ^]^

Glycogen drives glycolysis, and cell‐intrinsic glycogen metabolism supports early glycolytic reprogramming.^[^ ^21^ ^]^ Elevated GS and glycogen are associated with increased glycolysis during ischemia^[^ ^22^ ^]^ and compensatorily protective in anticipation of severe cardiac stress.^[^ ^23^ ^]^ Mobilizing heart glycogen to support glycolysis and produce ATP is needed for the intense contraction of the heart.^[^ ^24^ ^]^ Glycogen also provides succinate, an intermediate metabolite of canonical Krebs cycle activity, partly through glycolysis, further facilitating the mitochondrial respiratory pathway.^[^ ^25^ ^]^ In addition, cigarette smoke exposure‐induced mitochondrial dysfunction and heart damage are associated with changes in glycogen metabolism.^[^ ^26^ ^]^ A double substrate cycle operates between triose‐phosphates, glucose 6‐phosphate and glycogen influence mitochondrial acetyl‐CoA turnover in perfused rat hearts.^[^ ^27^ ^]^ Collectively, it can be speculated that glycogen driven ATP production and metabolic switches meet energy demand under metabolic stress.

Energy regulatory protein alpha‐1 acid glycoprotein (AAG) exerts various biological activities, including drug/ligand binding and disease markers.^[^ ^28^, ^29^, ^30^, ^31^ ^]^ AAG can interact with the skeletal muscle to reduce glucose oxidation, but it is not the result of an effect on glucose transport.^[^ ^32^ ^]^ However, the specific role of AAG in CF especially the jellyfish toxin‐induced one, and its potential therapeutic relevance remain largely unexplored.

Thus, we speculate whether AAG could alleviate jellyfish venom‐induced CF through its role in regulating glycogen metabolism, which could be a potential therapeutic target.

## 2 MATERIALS AND METHODS

### 2.1 Reagents

AAG and Cedilanid were purchased from Sigma (St. Louis, USA). Antibodies specific to AAG, PGC‐1α, CCL3, CCL4, CCL5 and CCL7 were obtained from BioWorld, Proteintech, Immunoway and cell signaling separately. Anti‐GAPDH Mouse mAb (PTM‐5150) was ordered from Jingjie PTM BIO. The Glycogen Assay Kit was ordered from Biovision (Solarbio, Guangzhou). The ATP Assay kit was from Sigma (St. Louis, USA).

### 2.2 Jellyfish venom extraction and preparation

Jellyfish were collected from coastal waters near Qingdao (China). Nematocysts were isolated from excised jellyfish tentacles, stirred continuously at 4°C for 72 h, and then filtered through a 200‐mesh sieve. The filtrate is centrifuged at 4°C, 1000 g for 15 min. The supernatant is taken and dialyzed overnight in 1 × PBS to obtain tentacle extracts (TE).

### 2.3 Human sample collection

Blood was obtained from jellyfish stings‐induced CF patient, and three healthy human donors without any heart disease. Cardiac ultrasound examination showed that the left ventricular wall thickens and the pulsation generally weakens, with reduced left ventricular systolic function and normal diastolic function. The patient's ejection fraction (EF) is less than 40%. A clinical trial (AF‐HEC‐054) was approved by the institutional ethics committee of PLA Naval Medical Center, Naval Medical University (Second Military Medical University). Informed consent was obtained from all enrolled patients and healthy controls.

### 2.4 Animals

C57 mice initially weighing 18–22 g, were provided by SIPPR/BK Laboratory Animals, Shanghai, China. AAG knockout mice were purchased from Cyagen Biosciences, Inc. The animals were housed under 12–12 h light dark cycle and allowed free access to standard laboratory food and water. All experimental and surgical procedures were undertaken in accordance with the animal protocol specifically for this study, approved by the Scientific Investigation Board of PLA Naval Medical Center, Naval Medical University (Second Military Medical University) (No.NMC2023011).

### 2.5 Processing scRNA‐seq data in clinic samples

Raw data generated with the 10× Genomics platform was aligned to the reference genome using Cell Ranger software (v7.0.1) to obtain the unique molecular identified (UMI) matrix, which was further imported into R (v4.2.2) and processed with the Seurat package (v4.3.2). Cells with a detected gene number <200 or >5000 or a high mitochondrial transcript ratio (>10%) were excluded. The top 30 principal components (PCs) were used for cluster analysis. Cell type was annotated by the SingleR package (v1.2.4) and then checked manually.

### 2.6 Echocardiography in conscious mice

A high‐resolution murine echo machine (Esaote MyLab One/Touch, Italy) was used. Mice were anesthetized with 2.5% isoflurane, maintained under anesthesia with 2.0% isoflurane, and examined. A long axis two‐dimensional image‐guided M‐mode 22 MHz view of the left ventricle (LV) was acquired and stored digitally. LV dimensions at diastole and systole (LVDd, LVDs) and heart rate (HR) were measured digitally from the M‐mode tracings. Teichholz formula calculates left ventricular end‐diastolic volume (LVEDV) and left ventricular end‐systolic volume (LVESV). Ejection fraction (EF%) = (LVEDV − LVESV) / LVEDV × 100% and Fractional shortening (FS%) = (LVIDd − LVIDs) / LVIDd × 100%. The observer who conducted the measurement was blinded to the treatment.

### 2.7 Hemodynamic measurement by intraventricular catheterization

The hemodynamic parameters were measured by the PowerLab data acquisition and analysis system (AD Instruments, ML870). The catheter was inserted into the left ventricle through the right carotid artery, and the AD Instruments PowerLab data acquisition and analysis system were connected at the other end. After stabilization (about 10 min), the left ventricular systolic pressure (LVSP) and maximum ascending rate of left ventricular pressure (Max dP/dt) were measured.

### 2.8 Western blotting

Cardiac homogenates and cardiac cells were homogenized on ice and lysed in a lysis buffer. Protein concentrations were measured using a BCA Protein Assay Kit (Novoprotein, Shanghai, China). Proteins were separated and transferred to a nitrocellulose (NC) membrane that was incubated with corresponding antibodies.

### 2.9 ELISA

Serum levels were detected by an ELISA kit (Shanghai Enzyme‐linked Biotechnology) according to the manufacturer instructions.

### 2.10 Glycogen and ATP detection

Glycogen and ATP were extracted and determined with the Glycogen Assay Kit and the ATP Assay Kit respectively.

### 2.11 Hematoxylin‐eosin (HE) staining and quantification

Heart tissues were fixed in 4% paraformaldehyde. Thereafter, fixed hearts were dehydrated, embedded in paraffin, and subsequently cut transversely into 4‐μm‐thick sections for H&E to measure cardiac tissue morphology. Images were captured using the Leica Microsystem, Germany.

### 2.12 Wheat germ agglutinin (WGA) and masson staining

WGA staining was used to measure myocardial hypertrophy. To histologically assess cardiac fibrosis, Masson's trichrome staining was performed on the tissue sections. All the stainings were captured, analyzed and quantified using the Leica Microsystem, Germany.

### 2.13 Human induced pluripotent stem cell‐derived cardiomyocytes (iPSC‐CMs) culture

Human iPSC‐CMs were purchased from Cosmos Biotechnological Co., Ltd. (Nanjing, China). The cells were seeded on 1% fibronectin pre‐coated optical bottom plates and were allowed to adhere for 48–72 h prior to replacement with cardiac maintenance medium.

### 2.14 Mitochondria isolation

Mitochondria were isolated from human iPSC‐CMs using a mitochondria isolation kit (Beyotime, Shanghai, China).

### 2.15 Seahorse metabolic assay

Seahorse XF96 Extracellular Flux Analyzer was used to measure extracellular acidification rate (ECAR, an index of glycolysis, mPH min^−1^) and real‐time ATP production (both mitoOCR and glycoPER, pmol min^−1^) in XF96 well plates (3 × 10^4^ cells/well) as per Installation and Operation Manual from Seahorse Bioscience (North Billerica, MA, USA). Cells were washed with Seahorse assay media (300 mg L^−1^ l‐glutamine, 2000 mg L^−1^ d‐glucose) and incubated in a CO_2_‐free incubator for 1 h. The value is obtained by Agilent wave analysis software and then exported to GraphPad for calculation.

### 2.16 Transmission electron microscopy

The sample was first double‐fixed with 2.5% glutaraldehyde and 1% OsO_4_, and dehydrated by a graded series of ethanol and acetone. The specimen was embedded in a mixture of absolute acetone and the final Spurr resin mixture, sectioned in the LEICA EM UC7 ultratome, stained with uranyl acetate and alkaline lead citrate and observed in the Hitachi HT7800 TEM.

### 2.17 Frozen tissue nuclear dissociation and 10× library preparation for snRNA‐seq

Mice hearts were harvested and frozen after sacrificing them. Frozen tissue samples were cut into pieces <0.5 cm and homogenized using a glass Dounce tissue grinder (Sigma, cat. no. D8938). Single cells were captured in droplet emulsions using the GemCode Single‐Cell Instrument (10× Genomics), and scRNA‐seq libraries were constructed as per the 10x Genomics protocol using the GemCode Single‐Cell 3′ Gel Bead and Library V2 Kit. The samples were diluted in PBS with 2% FBS to a concentration of 1000 cells per μL. Amplified cDNA and final libraries were evaluated on a fragment analyzer using a High Sensitivity next generation sequence (NGS) Analysis Kit (Advanced Analytical). Equal volumes of 16 libraries were pooled for sequencing on the NovaSeq 6000 Sequencing System (Illumina).

### 2.18 Differentially expressed genes (DEGs) analysis (Seurat) and UCell gene set scoring

To identify differentially expressed genes (DEGs), we used the Seurat FindMarkers function based on the Wilcoxon rank sum test with default parameters and selected the genes with an average log (fold change) value greater than 0.25 as DEGs. Adjusted *p*‐value was calculated by Bonferroni correction, and the value of 0.05 was used as the criterion to evaluate the statistical significance. Gene set scoring was performed using the R package UCell v 1.1.0.

### 2.19 Spatial transcription tissue collection and library preparation

Frozen samples were embedded in optimal cutting temperature compound (TissueTek), and cryosectioned into 10 μm slices at −10°C. Sections were placed on a chilled CytAssit Spatial Gene Expression slide (10× Genomics), and adhered by warming the back of the slide. cDNA libraries were generated using the CytAssit Spatial Gene Expression slide & Reagent Kit according to the manufacturer's instructions (10X Genomics), and sequenced on a NovaSeq 6000 system (Illumina).

### 2.20 Statistics

All data are presented as the mean ± SEM (Min to Max) unless otherwise stated and analyzed with GraphPad Prism 8.0 software. For data following a normal distribution, two‐sided unpaired Student's *t*‐tests (two‐group comparisons) or one‐way ANOVA with Tukey's post hoc test (three or more groups) were used. In analyses involving two factors, a two‐way ANOVA with Tukey's post hoc test for pairwise comparisons was applied. Repeated measure results were analyzed by a two‐way repeated measures ANOVA followed by a Bonferroni post hoc test. All experiments were replicated at least three times independently. Differences were considered significant at *p* < 0.05.

## 3 RESULTS

### 3.1 AAG expression is aberrantly expressed in failing human and murine samples

**FIGURE 1:**
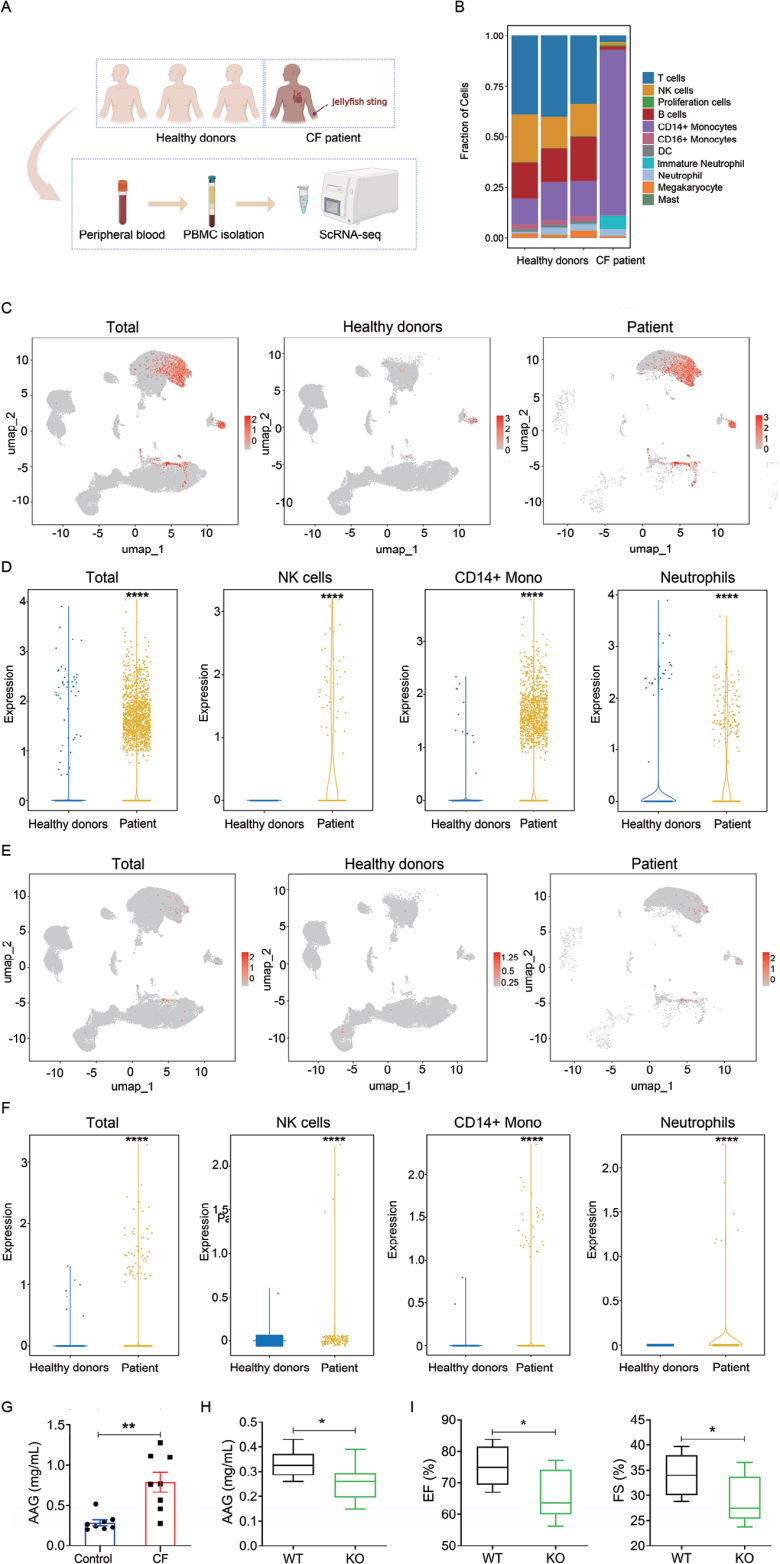
AAG is involved in the development of jellyfish stings‐induced CF. (A) Schematic representation of the single‐cell RNA‐sequence (scRNA‐seq) strategy. (B) Unbiased clustering from all four samples identifies 11 major cell types. (C) Uniform manifold approximation and projection (UMAP) plots show the expression of AAG1. (D) Violin plots show the distribution of normalized expression levels of AAG1 genes in total, NK cells, CD14+ monocytes and neutrophils. (E) UMAP plots show the expression of AAG2. (F) Violin plots show the distribution of normalized expression levels of AAG2 genes in total, NK cells, CD14+ monocytes and neutrophils. (G,H) The expression of serum AAG was measured by ELISA in jellyfish venom‐induced CF (G) and AAG KO mice (H). (I) AAG KO mice were more vulnerable to jellyfish venom‐induced cardiac dysfunction compared to littermates wild type (WT) mice, as shown by decreased ejection fraction (EF) and fractional shortening (FS). Data are presented as means ± SEM and analyzed by Student's *t*‐test (G–I). *p < 0.05, **p < 0.01, ****p < 0.0001. CF, cardiac failure; DC, dendritic cell; Mono, monocytes; NK, natural killer.

We performed single‐cell RNA sequencing on peripheral blood mononuclear cells (PBMCs) from the jellyfish stings‐induced CF patient and three healthy controls (HC). After standard data processing and quality control procedures, we obtained transcriptomic profiles for eleven major distinct cell clusters, including T cells, B cells, natural killer (NK) cells, CD14+ monocytes, CD16+ monocytes, dendritic cells (DC), neutrophils, immature neutrophils, megakaryocytes, masts and proliferation cells, were identified (Figure 1A,B). AAG1 was mainly expressed in CD14+ monocytes, neutrophils, and NK cells in the patient group, while it was only partially expressed in neutrophils in the HC group. The expression level of AAG1 in CD14+ monocytes, NK cells, and immature neutrophils was higher in the patient group than in the HC group (Figure 1C,D). The expression level of AAG2 is relatively low in PBMC, mainly in CD14+ monocytes, neutrophils, and NK cells in the patient group, while only sporadic cells are expressed in HC. The expression level of AAG2 in CD14+ monocytes, NK cells, and neutrophils in the patient group was significantly higher than that in the HC group (Figure 1E,F).

To clarify the relationship between AAG and CF, we examined AAG expression in jellyfish venom‐induced CF mice. AAG levels in sera significantly increased in CF mice after jellyfish venom administration (Figure 1G).

### 3.2 Deletion of AAG exacerbates CF

We next investigated the role of AAG in jellyfish venom‐induced CF. A loss‐of‐function approach was applied to determine the endogenous AAG. We selected 8‐week‐old male AAG−/− mice (Knockout, KO) and AAG+/+ mice (Wild type, WT) from the same littermate without disease model induction. AAG level was detected to confirm AAG knockout (Figure 1H), and echocardiography revealed reduced systolic function (EF and FS) in KO hearts (Figure 1I). Thus, the EF and FS of AAG−/− mice were significantly lower than those in the AAG+/+ group in physiological status, demonstrating poor cardiac function in vivo in the absence of AAG. Collectively, these findings suggest that deletion of AAG exacerbates CF progression, which prompted us to further investigate the potential pathological significance of the AAG action.

### 3.3 Endogenous AAG overexpression attenuates cardiac dysfunction

**FIGURE 2:**
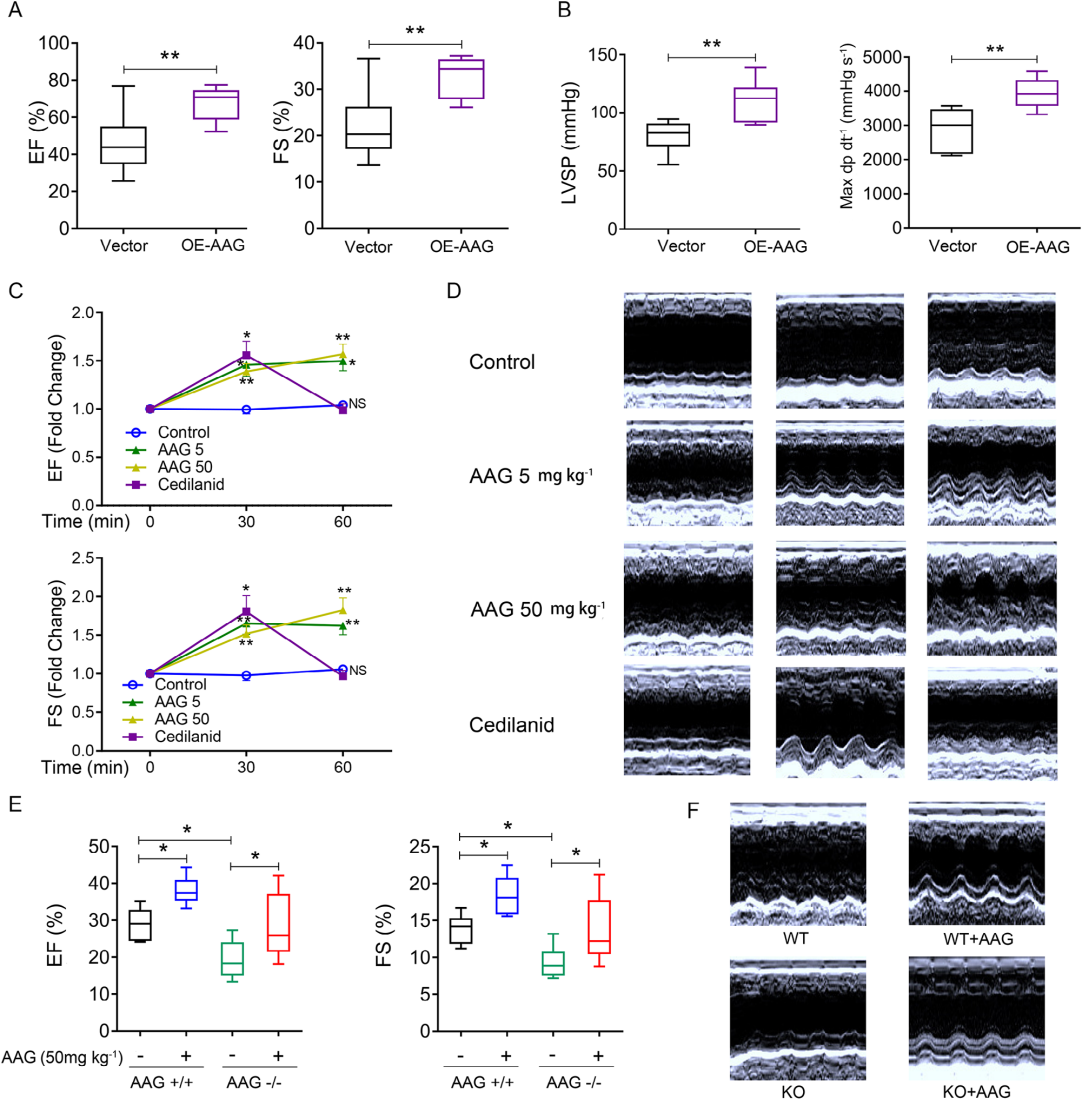
Endogenous and exogenous AAG could significantly improve cardiac function in jellyfish venom‐induced CF model rats and mice, respectively. (A,B) Lentivirus transfection AAG overexpression attenuated cardiac function and hemodynamic parameters decline in jellyfish venom‐induced CF. SD rat model was made, and AAG overexpression lentivirus (OE‐AAG) was injected into the myocardium. Cardiac function (A) EF, FS, and hemodynamic parameters (B) LVSP, Max dP/dt were analyzed. *N* = 8 in both groups. Data are presented as means ± SEM and analyzed by the Student's *t*‐test. (C,D) Mice were tail‐vein injected with AAG (5, 50 mg kg^−1^), cedilanid (positive control, 0.5 mg kg^−1^) or normal saline (control). Serial echography evaluates the function of model murine hearts. EF and FS were monitored for 0, 30, 60 min consecutively after administration by long‐axis 2‐dimensional M‐mode echocardiography. *N* = 6 per group. Data are presented as means ± SEM, and analyzed by a two‐way repeated measures ANOVA followed by Bonferroni post‐hoc test. (E,F) AAG administration rescued the lowered EF and FS in KO mice. Data are presented as means ± SEM and analyzed by ANOVA. *p < 0.05, **p < 0.01, NS denotes no significance. EF, ejection fraction; FS, fractional shortening; LVSP, left ventricular systolic pressure; Max dP/dt, maximum ascending rate of left ventricular pressure.

We next explored whether endogenous AAG overexpression attenuated or even prevented cardiac dysfunction progression. Sprague Daw (SD) rat jellyfish venom‐induced CF model was made, and AAG overexpression (OE) lentivirus (OE‐AAG) was injected into the myocardium at the same time. Cardiac function was detected by echocardiography. Vector control rats showed a decline in cardiac function, and this trend was significantly attenuated in OE‐AAG rats (Figure 2A). The hemodynamic parameters were also detected by the AD Instruments Powerlab data analysis system. The left ventricular systolic pressure (LVSP) and the maximum rise rate of left ventricular pressure (Max dP/dt) in the OE‐AAG group were significantly higher than that in the vector control group, that is, the decline was attenuated under AAG overexpression (Figure 2B).

### 3.4 Exogenous AAG administration significantly improves cardiac function

We next observed the effect of exogenous AAG on jellyfish venom‐induced CF model mice. Echocardiography detected cardiac function and confirmed CF (EF < 60%) in mice. Mice were tail‐vein injected with AAG (5, 50 mg kg^−1^), cedilanid (positive control, 0.5 mg kg^−1^) or normal saline (control), and continuously monitored by long‐axis 2‐dimensional M‐mode echocardiography. At 60 min after administration, left ventricular EF and FS were remarkably elevated significantly. The effect of the positive control drug cedilanid was the most prominent at 30 min after administration, but it returned to the baseline level at 60 min (Figure 2C,D). Moreover, a jellyfish venom‐induced CF model was developed in AAG+/+ and AAG−/− mice. At the functional level, AAG KO mice showed a dramatic decline in cardiac systolic performance. AAG administration rescued the lowered EF and FS in KO mice (Figure 2E,F). Taken together, these findings suggest that maintaining AAG expression may exert a beneficial effect to attenuate or even prevent CF progression.

### 3.5 AAG significantly improves cardiac metabolic adaptation to CF

**FIGURE 3:**
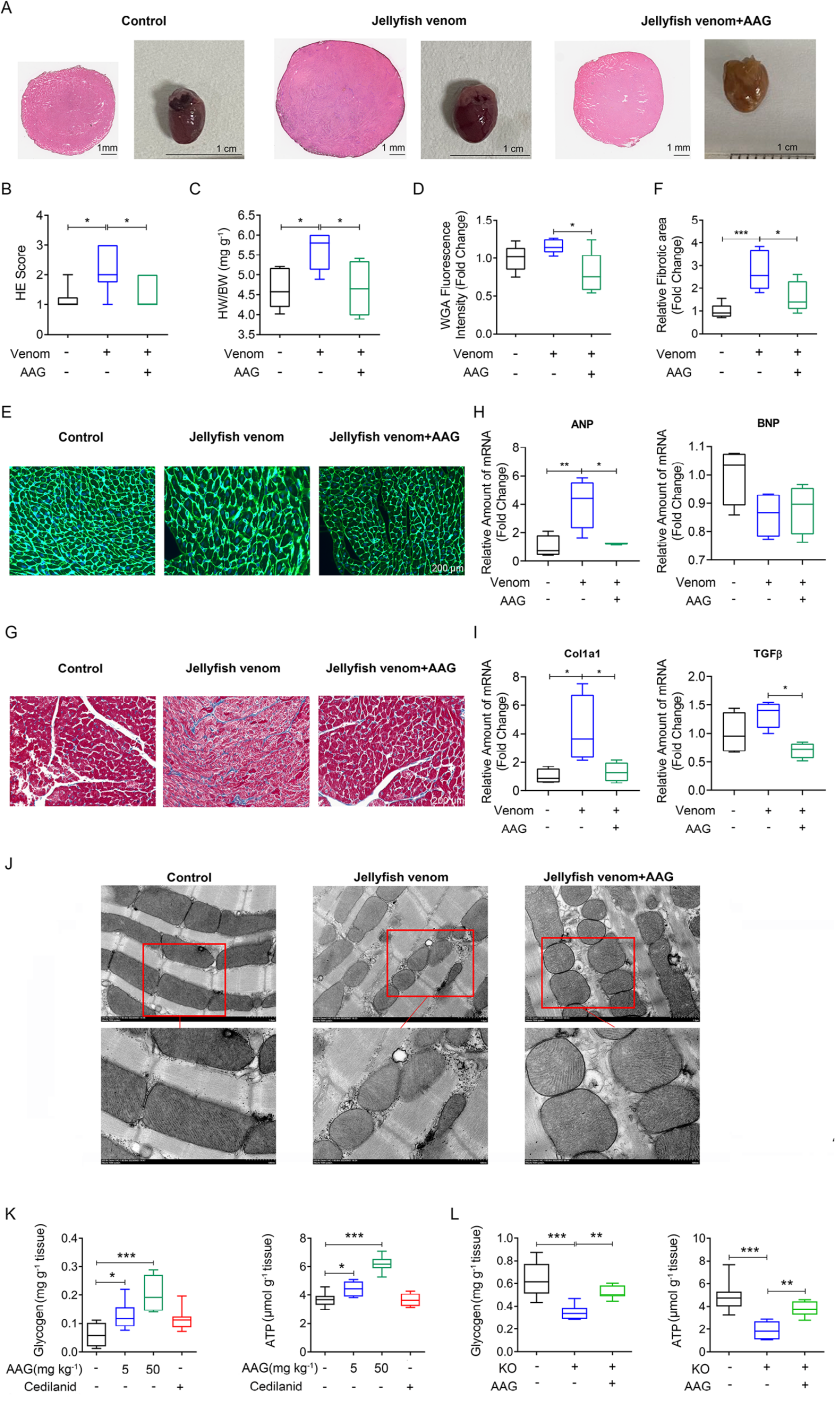
AAG ameliorated pathological manifestations of cardiac failure and promoted glycogen‐ATP in vivo. (A–C) Hematoxylin & eosin (HE) staining and the heart weight (HW) to body weight (BW) ratio in control group, jellyfish venom model group, and jellyfish venom with AAG administration group. *N* = 6 per group. Data are presented as means ± SEM and analyzed by one‐way ANOVA. Representative HE images (scale: 1 mm) and anatomic images (Scale: 1 cm) (A). HE score quantification of images (B). The heart weight to body weight ratio (HW/BW) of mice in each group (C). (D,E) Wheat germ agglutinin (WGA) staining of cardiomyocyte cross‐sectional area and corresponding fluorescence intensity quantification. The cardiomyocyte size was illustrated by green WGA staining. Scale: 200 μm. (F, G) Masson's trichrome staining with fibrotic regions (blue) and corresponding quantification of blue‐staining relative fibrotic area. Scale: 200 μm. (H) Q‐PCR was performed to analyze the mRNA levels of cardiac hypertrophy markers (ANP, BNP). (I) Q‐PCR was performed to analyze the mRNA levels of fibrosis markers (Col1a1, TGF‐β). (J) Transmission electron microscopic (TEM) images. Scale bar: 1 μm (upper panel), 500 nm (lower panel). (K) Glycogen and ATP level in murine cardiac tissues described as Figure 2C. (L) AAG administration rescued cardiac glycogen and ATP level decline in cardiac tissues of KO mice. *p < 0.05, **p < 0.01, ***p < 0.001. ANP, atrial natriuretic peptide; BNP, Brain natriuretic peptide; Col1α1, Collagen type 1 alpha 1 chain; HE, hematoxylin‐eosin staining; TGFβ, Transforming growth factor‐beta; WGA, Wheat germ agglutinin.

Left ventricular chamber size in jellyfish venom‐induced CF model mice was significantly more dilated, with elevated HE score and heart weight/body weight (HW/BW) ratios, which were reversed in the model group with CF mice administered AAG (Figure 3A–C). Cardiomyocyte cross‐sectional area stained with wheat germ agglutinin (WGA) was used to analyze cardiac hypertrophy, which was significantly greater in model mice compared with control hearts (Figure 3D,E). Excessive collagen deposition was stained with Masson trichrome in model murine hearts, indicating elevated cardiac fibrosis, a classic feature of pathologic cardiac remodeling (Figure 3F,G). Additionally, q‐PCR was performed to analyze the mRNA levels of genes related to cardiac hypertrophy and fibrosis. Indeed, we found highly expressed hypertrophy marker atrial natriuretic peptides (ANP) in jellyfish venom‐induced CF model murine cardiomyocytes. Also, the fibrosis marker Collagen type 1 alpha 1 chain (Col1a1) was highly expressed in a jellyfish venom‐induced CF model murine heart. AAG administration significantly reversed raised cardiac hypertrophy and fibrosis marker expression (Figure 3H,I). AAG administration ameliorated cardiac hypertrophy and fibrosis characterized by lower WGA and Masson level (Figure 3D–I). Transmission electron microscopy (TEM) imaging of cardiac mitochondria is a valuable technique for examining the ultrastructural changes within these organelles in various groups. In the control group, TEM images of cardiac mitochondria revealed a well‐preserved and organized structure, indicating efficient energy production and normal mitochondrial function. In the jellyfish venom‐induced CF model group, TEM images demonstrated notable alterations in mitochondrial ultrastructure. One of the prominent findings was the presence of swollen and disrupted mitochondria. These changes are indicative of mitochondrial dysfunction and may suggest impaired OXPHOS, which can lead to reduced energy production. Additionally, the loss of well‐defined cristae and a reduction in electron density within the matrix further emphasized the detrimental impact of the jellyfish venom‐induced CF model. The observed structural abnormalities are consistent with the development of cardiac dysfunction and CF. AAG treatment reduced mitochondrial swelling and partially restored cristae structure, suggesting that it may have a protective effect on mitochondrial integrity and function (Figure 3J).

**FIGURE 4:**
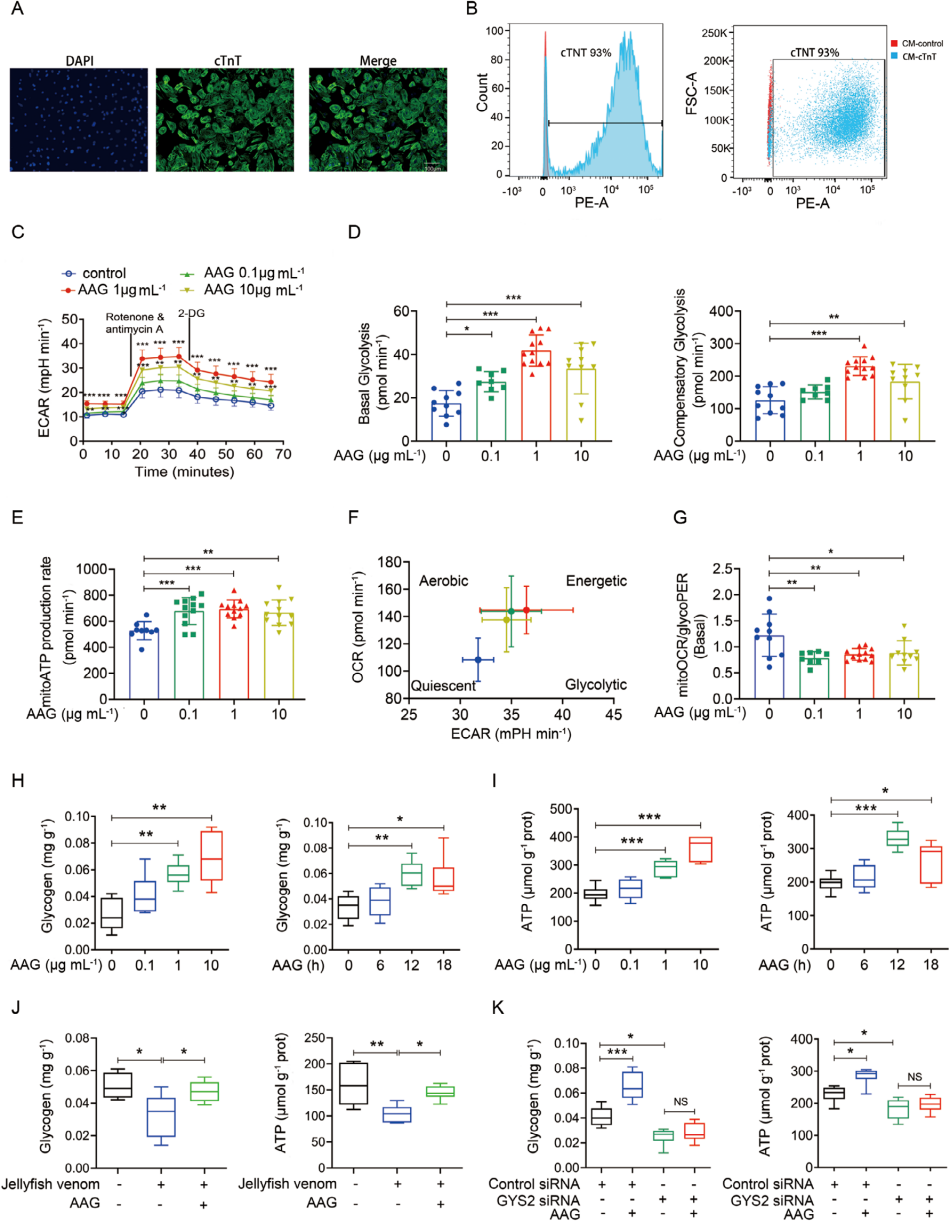
AAG promoted glycolytic/mitochondrial metabolic switches through glycogen‐driven ATP production in vitro human iPSC‐CMs. (A,B) Purity detection of human iPSC‐CMs. Representative images of cultured cardiomyocytes stained for cardiac Troponin T (cTnT, green) and nuclei (blue) (A). Human iPSC‐CMs were highly positive for cTnT. The cardiomyocytes were labeled for cTnT up to 93% to ensure purity using Flow cytometry analysis (B). (C–G) Detection of mitochondrial and glycolytic modulators using the Seahorse XF Real‐Time ATP Rate Assay and Seahorse XF Glycolytic Rate Assay. Human iPSC‐CMs were treated with the vehicle control or AAG (0.1, 1, 10 μg mL^−1^). ECAR profiles of Glycolytic Rate Assay (C). Quantification of basal glycolysis and compensatory glycolysis (D). Mitochondrial ATP production rate (E). Energetic map tested charting energetic phenotype switches between mitochondrial ATP and glycolysis‐generated ATP. OCR versus ECAR (F). mitoOCR/glycoPER (G). All Seahorse data shown are compared using 9–12 replicates. (H) AAG administration enhanced glycogen level in human iPSC‐CMs dose‐ and time‐ dependently. (I) AAG administration enhanced ATP level in human iPSC‐CMs dose‐ and time‐dependently. (J) AAG‐enhanced glycogen and ATP level in jellyfish venom stimulation modeled human iPSC‐CMs separately. (K) Glycogen and ATP level decreased significantly after GSY2 silencing, and AAG could not increase glycogen and ATP content silenced by GSY2. *N* = 6 per group. Data are presented as means ± SEM, and analyzed by one‐way ANOVA (D‐K) or two‐way repeated measures ANOVA (C). ECAR, extracellular acidification rate; OCR, oxygen consumption rate; PER, proton efflux rate.

Human iPSC‐CMs provide an unlimited patient‐specific cell source for CF disease modeling, cardiotoxicity screening, and cardiac repairment.^[^ ^33^, ^34^ ^]^ We detected purity of human iPSC‐CMs. Human iPSC‐CMs were highly positive for cardiac Troponin T (cTnT), labeled for cTnT up to 93% to ensure purity using flow cytometry analysis (Figure 4A,B). Increased ETC complex activity suggested a potential enhancement of ATP production and metabolic adaptation in AAG‐treated cardiomyocytes. To corroborate this hypothesis, we used an extracellular flux analyzer to real‐time monitor ATP production and energetic phenotype switches in iPSC‐CMs. As expected, AAG treatment elevated glycolysis at both basal and maximal levels as evidenced by increased extracellular acidification rate (ECAR) using seahorse Xglycolytic rate assay (Figure 4C,D). AAG administration also dramatically improved OXPHOS as evidenced by the elevated mitoATP production rate (Figure 4E). The increase in both OXPHOS and glycolysis is also indicated in the energy map. AAG shifted mitoOCR/glycoPER towards glycolysis, which is also validated by the lowered energetic rate of mitoOCR/glycoPER (Figure 4F,G). These data demonstrate that AAG increased the proportion of glycolysis‐derived ATP to rely energetically primarily on glycolysis. Briefly, AAG increases both mitochondrial and glycolytic metabolism, particularly glycolysis.

### 3.6 AAG significantly improves glycogen‐driven ATP production

CF is characterized by changes in substrate utilization leading to a decline in ATP levels.^[^ ^35^, ^36^, ^37^ ^]^ The inability of ATP production and further reduced energy conversion in mechanical work drives contractile function loss, resulting in decreased cardiac function.^[^ ^38^, ^39^ ^]^ We next explored the effect of AAG administration on glycogen and ATP. In jellyfish venom‐induced CF model mice, AAG (5, 50 mg kg^−1^), cedilanid (positive control, 0.5 mg kg^−1^) or normal saline (control) were tail‐vein administered. After 60 min, cardiac glycogen and ATP content in AAG administration group were significantly higher than those in control group. Cedilanid (0.5 mg kg^−1^) did not increase ATP and glycogen content in the cardiac tissues of CF model mice (Figure 3K). Similarly, cardiac glycogen and ATP content in AAG KO mice were significantly lower than those in wild‐type group. AAG administration rescued cardiac glycogen and ATP level decline in KO mice (Figure 3L).

In vitro, AAG elevated glycogen and ATP levels in a dose and time dependent manner in iPSC‐CMs (Figure 4H,I). Furthermore, we used jellyfish venom to construct the CF model in vitro separately. As expected, AAG administration significantly elevated glycogen and ATP level (Figure 4J). We further studied the effect of glycogen on ATP synthesis, investigating whether AAG significantly improves glycogen‐driven ATP production. RNA interference was used to silence GSY2, a key gene for glycogen synthesis. The results showed that glycogen content decreased significantly after GSY2 silencing in human iPSC‐CMs, and AAG could not increase glycogen content in human iPSC‐CMs silenced by GSY2. Meanwhile, ATP level decreased significantly after GSY2 silencing, and the effect of AAG on increasing ATP content disappeared with GSY2 silencing (Figure 4K), suggesting that glycogen is essential for ATP production. Collectively, glycogen may be an important intermediate link in the promotion of ATP production by AAG.

### 3.7 Single‐nucleus and spatial transcriptomic sequencing of jellyfish‐induced murine hearts with or without AAG administration

**FIGURE 5:**
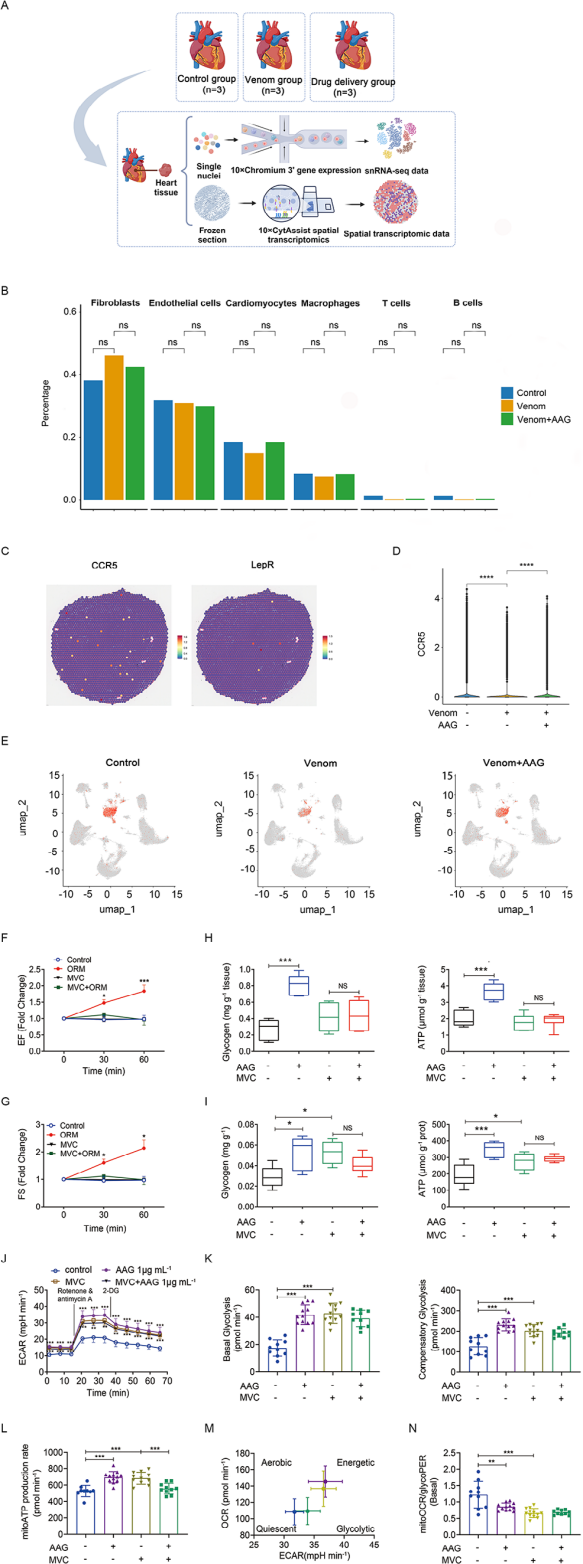
CCR5 mediates AAG's improving effects on cardiac function and metabolic switches. (A) Schematic representation of the overall experimental procedure. The cardiac tissues of control, jellyfish venom‐induced mice with or without AAG administration were collected for snRNA‐seq (*n* = 3; total 9 samples). (B) The proportions of each cell cluster between each group. (C) CCR5 was expressed and located, while LepR was barely expressed in cardiac murine tissues, assessed by spatial transcription technology. (D) Violin plot quantification of CCR5 expression in each group. (E) UMAP plots show the expression of CCR5. (F,G) Mice were intragastrically administered MVC 50 mg kg^−1^ (or saline), with or without AAG tail‐vein injected (50 mg kg^−1^). Serial echography evaluates the cardiac function (EF and FS) 0, 30, 60 min consecutively after administration. (H,I) In vivo murine cardiac tissues and in vitro human iPSC‐CMs, the increased cardiac glycogen and ATP content were abolished by CCR5 specific inhibitor MVC. (J,K) The increased glycolysis ECAR on AAG‐treated human iPSC‐CMs was abolished by MVC. ECAR profiles of Glycolytic Rate Assay (J). Quantification of basal glycolysis and compensatory glycolysis (K). (L) The increased mitoATP production rate on AAG‐treated human iPSC‐CMs was abolished by MVC. (M,N) The energetic phenotype switches and decreased mitoOCR/glycoPER on AAG‐treated human iPSC‐CMs were largely blocked by MVC. Human iPSC‐CMs were pre‐incubated with MVC for 3 h. *N* = 6 (F,G) or *N* = 9–12 (J–N) per group, and analyzed by two‐way repeated measures ANOVA followed by Bonferroni post‐hoc test (F,G,J) or ANOVA followed by TukeyAN post‐hoc test (D,H,I,K,L,N). *p < 0.05, **p < 0.01, ***p < 0.001, ****p < 0.0001, NS denotes no significance. ECAR, extracellular acidification rate; MVC, maraviroc; OCR, oxygen consumption rate; PER, proton efflux rate.

To obtain a comprehensive landscape of cardiomyocytes and immune cells after injury of the heart, we performed snRNA‐seq and spatial transcription technology. Male C57BL/6J mice aged 7 weeks were randomly divided into Control, Venom and AAG administration groups. A total of 93,143 cells captured after quality control filtering were integrated, clustered, and visualized in uniform manifold approximation and projection (UMAP) plots using Seurat v 3.1.2 (Figure 5A). In the Control group, Fibroblasts represented the largest cell population (38.2% of total cells) in the murine hearts, followed by Endothelial cells (31.9%), Cardiomyocytes (18.6%), Macrophages (8.4%), T cells (1.5%), and B cells (1.4%) (Figure 5B). All samples were sequenced individually.

We further verified and explored the mechanism in AAG therapy for CF using scRNA‐seq. The differentially expressed genes (DEGs) were screened with *p* value < 0.05 and |log_2_fold change| > 0.25. Comparing Control and Venom group, a total of 1349 DEGs were obtained, among which 541 genes were up‐regulated and 808 genes were down‐regulated. Comparing Venom and Venom+AAG group, a total of 351 DEGs were obtained, among which 238 genes were up‐regulated and 113 genes were down‐regulated.

Studies uncovered that AAG might bind to cellular surfaces. CCR5 receptors are also expressed on cardiomyocytes,^[^ ^40^ ^]^ and involved in cardiac remodeling and dysfunction under stress overload.^[^ ^41^ ^]^ We used spatial transcription technology, only to find CCR5 was expressed and located, while LepR was barely expressed in cardiac murine tissues (Figure 5C). Our sequencing data uncovered that CCR5 was mainly expressed on cardiomyocytes and macrophages. The expression level of CCR5 was lower in the Venom group than in the Control group, and AAG administration elevated CCR5 expression (Figure 5D,E).

### 3.8 CCR5 mediates AAG's promoting effect on cardiac function

We next asked whether CCR5 blockade affects the action of AAG on cardiac function. We then observed the effect of CCR5 on jellyfish venom‐induced CF model mice. We performed a reciprocal experiment in which CF model mice were first intragastrically administered with MVC (CCR5 specific inhibitor), then tail‐vein injected with AAG (50 mg kg^−1^). Serial echocardiographic measurements showed that EF and FS were significantly higher in the AAG group at 60 min compared with those in the control group, while EF and FS were maintained in mice pre‐administered with MVC (Figure 5F,G). Thus, the CCR5 receptor mediates AAG's promoting effect on cardiac function.

### 3.9 CCR5 mediates AAG's improving effect on glycogen‐driven ATP production and metabolic switches

We also asked whether CCR5 blockade affects the action of AAG on glycogen‐driven ATP production and metabolic switches accordingly. In vivo murine cardiac tissues and in vitro human iPSC‐CMs, the increased role of AAG in glycogen and ATP content was abolished by MVC (Figure 5H,I). Thus, CCR5 mediates AAG's improving effect on glycogen‐driven ATP production.

We then used an extracellular flux analyzer to real‐time monitor ATP production and energetic phenotype switches in human iPSC‐CMs pre‐incubated with CCR5 specific inhibitor MVC. In vitro, the elevated effect of AAG on glycolysis at both basal and maximal levels, as evidenced by increased ECAR was largely blocked by MVC (Figure 5J,K). Additionally, the improved effect of AAG on OXPHOS as evidenced by the elevated mitoATP production rate, was largely blocked by MVC (Figure 5L). The shifting role of AAG on mitoOCR/glycoPER towards glycolysis and the lowered energetic rate of mitoOCR/glycoPER were also attenuated by MVC (Figure 5M,N). Thus, CCR5 mediates AAG's improving effect on glycogen‐induced glycolysis/mitochondrial ATP production and metabolic switches.

### 3.10 The effect of AAG‐CCR5 was correlated with PGC‐1α genes

**FIGURE 6:**
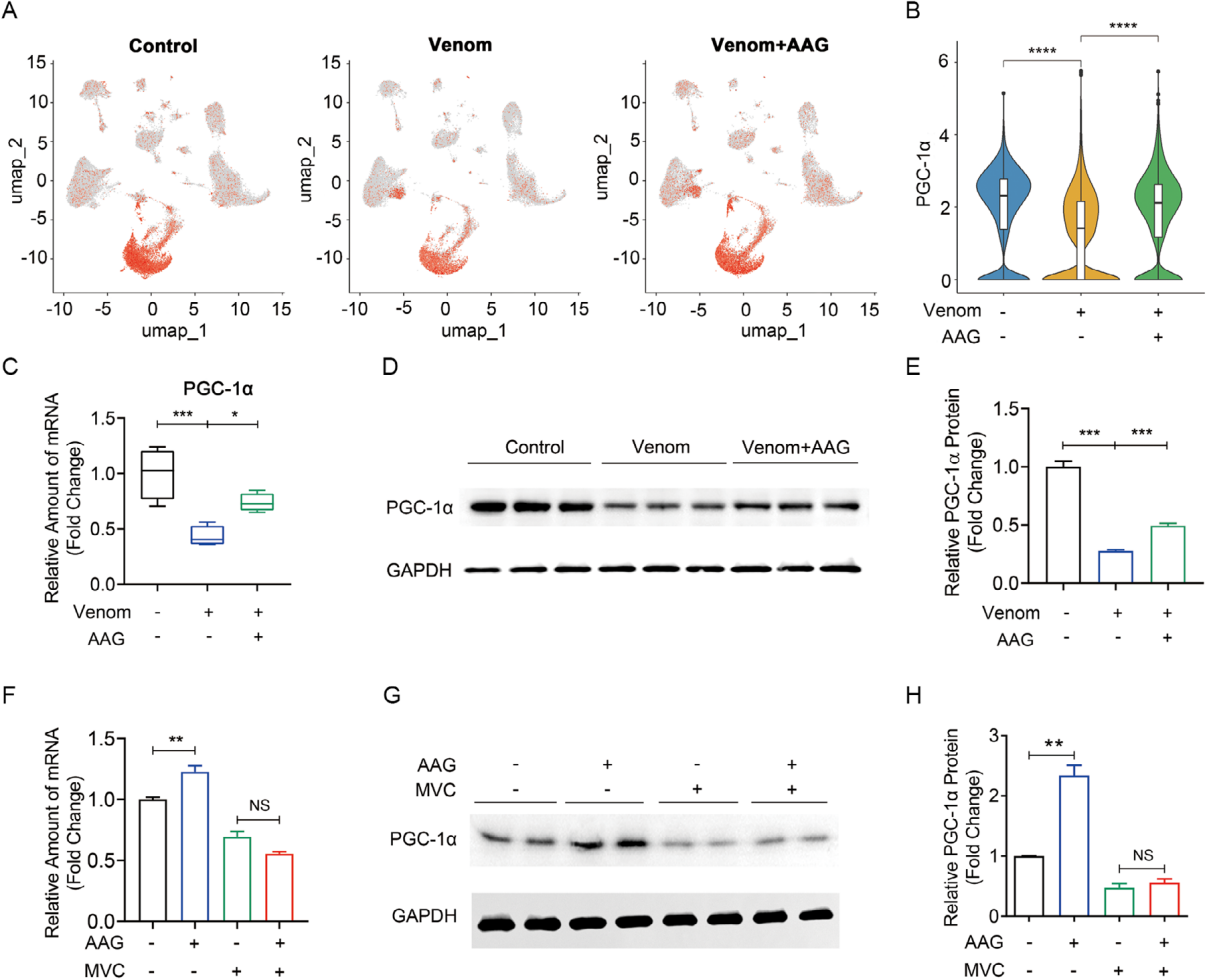
The promoting effect of AAG‐CCR5 was correlated with PGC‐1α. (A) UMAP plots show the expression of PGC‐1α. (B) Violin plot quantification of PGC‐1α expression in each group. (C) PGC‐1α mRNA level was validated by PCR. (D,E) PGC‐1α protein expression was validated by WB and corresponding quantification. (F–H) Mice were intragastrically administered MVC 50 mg kg^−1^ (or saline), with or without AAG tail‐vein injected (50 mg kg^−1^). The increased PGC‐1α mRNA (F) and protein expression (G,H) were abolished by MVC in murine cardiac tissues. *N* = 3–6 per group, and analyzed by ANOVA followed by Tukey's post‐hoc test. *p < 0.05, **p < 0.01, ***p < 0.001, ****p < 0.0001, NS denotes no significance. MVC, Maraviroc; PGC‐1α, Peroxisome proliferator‐activated receptor‐gamma coactivator‐1alpha.

In order to further explore the downstream signaling pathway, we screened some related genes that could regulate the process of cardiac glycogen metabolism, only to find Peroxisome proliferator activated receptor coactivator‐1α (PGC‐1α). PGC‐1α was mainly expressed in cardiomyocytes. The expression level of PGC‐1α was lower in the Venom group than in the Control group, and AAG administration elevated PGC‐1α expression (Figure 6A,B). Then the mRNA and protein expression of PGC‐1α were validated. The mRNA and protein expression of PGC‐1α in Venom group were decreased compared with that in normal control group, and AAG elevated the PGC‐1α expression (Figure 6C–E). Additionally, CCR5 specific inhibitor MVC abolished the elevated AAG's effect on PGC‐1α expression (Figure 6F–H). Thus, AAG elevated PGC‐1α expression at the transcriptional and translational level, which may be mediated by CCR5.

### 3.11 AAG significantly improves CCR5 receptor ligands

**FIGURE 7:**
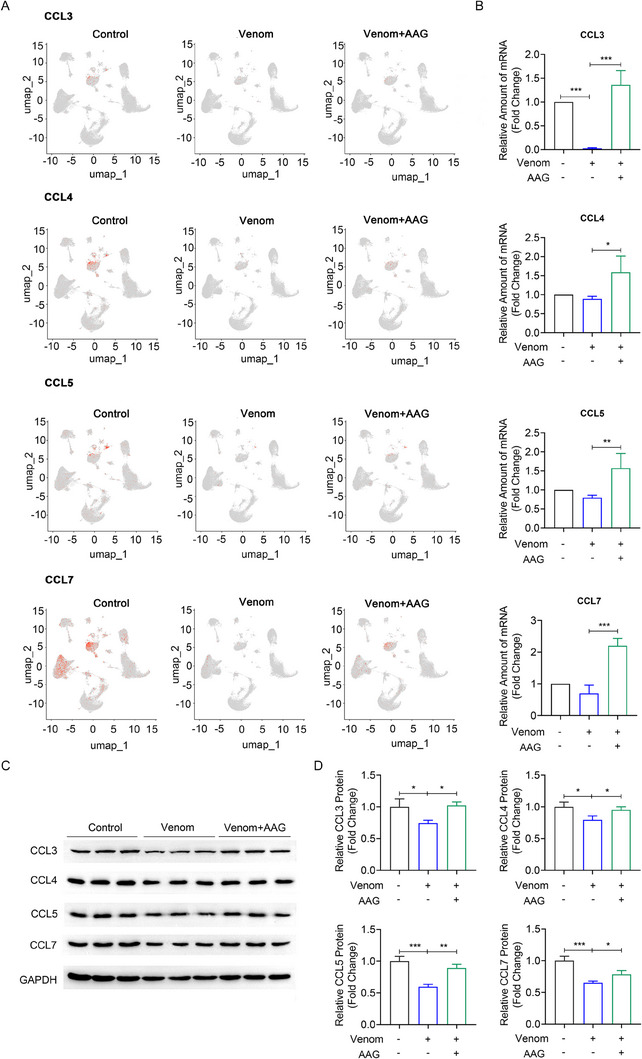
AAG significantly improves CCR5 receptor ligands. (A) UMAP plots show the expression of CCR5 receptor ligands CCL3, CCL4, CCL5 and CCL7 separately. (B) PCR detected normalized mRNA levels of CCR5 receptor ligands CCL3, CCL4, CCL5 and CCL7 separately. (C) WB detected normalized protein levels of CCR5 receptor ligands CCL3, CCL4, CCL5 and CCL7 separately. (D) Quantitative analysis of the western blot results. *N* = 3–5 per group. Data are presented as means ± SEM, and analyzed by one‐way ANOVA followed by a Tukeyan post‐hoc test. *p < 0.05, **p < 0.01, ***p < 0.001.

As we know, there are many ligands for CCR5 receptor, for example, CCL3, CCL4, CCL5, and CCL7. We further detected the effect of AAG on these CCR5 receptor ligands. They were mainly expressed on macrophages and T cells. UMAP plots, mRNA and protein levels were all lower in the Venom group than in the Control group, and AAG administration hugely elevated their expression (Figure 7), demonstrating AAG significantly improves CCR5 receptor ligands both at transcriptional and translational levels.

## 4 DISCUSSION

Man‐Tat Lau et al., have identified essential genes and cellular pathways involved in the jellyfish venom mechanisms triggering cell death.^[^ ^42^ ^]^ The function of glycogen varies in different tissues. It is reported that in adipose tissue, glycogen mainly drives browning to produce heat. While in cardiac tissue, glycogen mainly produces ATP as the main energy supply. Studies have reported that glycogen plays a crucial role in cardiometabolic stress caused by obesity, exercise, fasting, ischemia and other factors, and the ability to synthesize glycogen in myocardium is critical for normal heart development.^[^ ^42^, ^43^ ^]^ Glycogen metabolism is a CF‐induced key pathway, and is necessary for optimal glucose utilization, representing the targeted mechanism of metabolic adaptation.^[^ ^45^, ^46^, ^47^ ^]^ An article published in *Nature* reported that glycogen metabolism links glucose homeostasis to thermogenesis, which might have therapeutic benefits.^[^ ^43^ ^]^ Yoshihiko et al., reported that the lack of glycogen in the liver triggered the liver‐brain‐adipose neural axis, which promoted the fatty acids and glycerol release from white adipose tissue. Intriguingly, AAG promoted glycogen synthesis, further facilitating glycolysis.^[^ ^48^ ^]^

A recent report revealed that AAG has prognostic value in peripheral artery disease. Higher AAG levels are independently associated with the development of adverse peripheral artery disease‐related events.^[^ ^49^ ^]^ In mortality risk assessment for elderly patients, AAG may be superior to C‐reactive protein (CRP).^[^ ^50^ ^]^ To probe into the function of AAG in heart, we used AAG KO mice to induce metabolic dysregulation typically accompanied by CF. We showed here that the absence of AAG led to deterioration of cardiac function, and the aggravation of cardiac hypertrophy and fibrosis. The synthesis of ATP in AAG KO hearts was insufficient to meet increased demand. Hence, hearts from AAG KO mice were unable to normally perform the work of contraction, showing a similar CF phenotype. These data indicated that AAG was an attractive candidate for mediating cardiac metabolic alterations, since mice lacking AAG had reduced cardiac energy reserves and impaired cardiac contractility, and functional AAG was critical for ATP generation.

The inherent complexity of mitochondrial processes related to energy provision and redox homeostasis. AAG administration mitigates the detrimental effects of jellyfish toxins on depleted glycogen stores and impaired energy production in cardiomyocytes, leading to contractile dysfunction. AAG preserved glycogen levels and enhanced energy production, ultimately improving cellular contractility. That is, AAG administration ameliorated jellyfish toxin‐induced cardiac dysfunction, accompanied by increased glycogen content and restored energy production.

Mechanistically, CCR5, involved in cardiac remodeling and dysfunction under stress overload,^[^ ^41^ ^]^ may be implicated in the development and progression of CF.^[^ ^51^ ^]^ Research has shown that CCR5+ inflammatory cells and increased CCR5 ligands (CCL3, CCL4 and CCL5) may contribute to the recruitment of inflammatory cells to the inflamed tissue and their activation.^[^ ^52^, ^53^ ^]^ We uncovered that these CCR5 ligands were mainly expressed on macrophages and T cells. Moreover, AAG significantly improved CCR5 receptor ligands both at transcriptional and translational levels. Thus, AAG might contribute to the recruitment of inflammatory cells to the inflamed tissue and their activation.

Previous studies have reported the involvement of CCR5 in cardiac remodeling through the activation of immune responses.^[^ ^41^, ^54^ ^]^ However, our findings suggest that CCR5 inhibitor MVC did not impact CF in our experimental model. The involvement of immune cells, such as macrophages and T cells, in CF is multifaceted, and the contribution of individual immune cell subsets may vary depending on the specific pathological conditions. In our model, CCR5 expression was low in the cardiac failure model group, so the effect of CCR5 inhibition may not be obvious enough. Notably, MVC administration had a protective effect in human iPSC‐CMs experiments without jellyfish venom stimulation.

PGC‐1α is an attractive target for mitochondrial homeostasis and a central regulator of heart metabolism,^[^ ^55^ ^]^ increasing cardiac mitochondrial respiration. PGC‐1α absence causes a decrease in available ATP supply, leading to impaired cardiac contractility. Overexpression of PGC‐1α in cultured cardiomyocytes and murine myocardium upregulates a large number of genes important for tricarboxylic acid cycle (TCA) and respiratory chain complex.^[^ ^56^, ^57^, ^58^ ^]^ Thus, our study highlights the pivotal role of AAG in enhancing glycogen and ATP production. AAG elevates PGC‐1α expression via CCR5, strengthening cardiac function.

Cardiotoxicity is responsible for the venom's lethality, including a remarkable elevation in ST and T wave inversion in the electrocardiogram, which indicated that an acute cardiac infarction occurred. In this study, we present compelling evidence in vitro and in vivo, highlighting the potential of AAG as a modulator of glycogen‐driven ATP production that can attenuate the detrimental effects of jellyfish venom on CF. By deciphering the intricate interplay between AAG, glycogen metabolism and cardiac function, we can potentially identify AAG as a promising therapeutic agent for jellyfish venom‐induced CF.

Our study suggests that AAG plays a crucial role in regulating glycogen‐driven ATP production, promoting glycolytic/mitochondrial metabolic switches. AAG administration alleviates the detrimental effects of jellyfish toxins on cardiac function by preserving glycogen stores and enhancing energy production. These findings highlight the potential of AAG as a novel approach in the management of jellyfish toxin‐induced CF. Importantly, AAG could be prepared from blood products and present in the human body, which has a promising clinical transformation prospect and good safety. Further investigations are warranted to explore the precise mechanisms involved and assess the long‐term efficacy of AAG as a therapeutic intervention.

## CONFLICT OF INTEREST STATEMENT

The authors declare no conflicts of interest.

## Data Availability

The data that support the findings of this study are available on request from the corresponding author. The data are not publicly available due to privacy or ethical restrictions.
